# The Development of Predictive Nomogram of Recurrence for Patients With Endometrioma After Cystectomy Who Were Younger Than 45 Years Old and Received Postoperative Therapy

**DOI:** 10.3389/fmed.2022.872481

**Published:** 2022-06-09

**Authors:** Zhiyue Gu, Xiaoyan Li, Jinghua Shi, Yushi Wu, Jing Zhang, Chenyu Zhang, Hailan Yan, Jinhua Leng

**Affiliations:** ^1^Department of Obstetrics and Gynecology, Peking Union Medical College Hospital, Chinese Academy of Medical Sciences and Peking Union Medical College, Beijing, China; ^2^National Clinical Research Center for Obstetric and Gynecologic Diseases, Beijing, China

**Keywords:** endometriosis, endometrioma, nomogram, recurrence, predictive model

## Abstract

**Objective:**

This study aimed to establish an effective prognostic nomogram for the postoperative recurrence of endometrioma or endometriosis-related pain for patients with endometrioma after long-term follow-up, who were younger than 45 years old and received postoperative therapy.

**Methods:**

The predictive nomogram was based on 323 patients who underwent cystectomy for endometrioma at Perking Union Medical College Hospital from January 2009 to April 2013, and the last follow-up occurred in September 2018. We collected information on all included patients, including preoperative data, intraoperative data, and long-term follow-up data after surgery. The Cox proportional hazards regression model was used to evaluate the prognostic effects of multiple clinical parameters on recurrence. The survival curve was depicted based on Kaplan-Meier method and compared by log-rank method. The Index of concordance (C-index) and calibration curves were used to access the discrimination ability and predictive accuracy of the nomogram respectively, and the results were further validated via bootstrap resampling. In addition, calculating the area under the curve (AUC) via risk scores of patients aimed to further access the prediction ability of the model.

**Results:**

On multivariate analysis of derivation cohort, independent factors for recurrence such as dysmenorrhea degree, sum of both cyst diameters, presence of adenomyosis, and other essential factors for recurrence such as age at surgery, presence of uterine fibroids were all selected into the nomogram. The C-index of the nomogram for predicting recurrence was 0.683 (95% CI, 0.610- 0.755). The calibration curve for probability of recurrence for 7 years and 9 years showed great agreement between prediction by nomogram and actual observation. Furthermore, the AUCs of risk score for 7-year and 9-year were 0.680 and 0.790 respectively.

**Conclusion:**

This research tried to develop the predictive nomogram of recurrence for patients with endometrioma after cystectomy. The C-index and calibration curve of nomogram, as well as the AUC of the nomogram was potential to predict the recurrence probability. In addition, this predictive nomogram needs external data sets to further validate its prognostic accuracy in the future.

## Introduction

As a prevalent gynecological disease, endometriosis (EM) approximately debilitated 6–10% women of reproductive age (1). Endometriosis usually occurs in the pelvic cavity and can be divided into three subtypes: superficial peritoneal endometriosis (PEM), deep endometriosis (DE), and ovarian endometriosis (endometrioma) (2, 3). Endometrioma accounts for 17–44% of EM and is often associated with infertility (4). The main treatments of endometrioma include medical and surgical management, and the cystectomy of endometrioma is the most prevalent procedure. However, patients who completed conservative surgery still have a certain probability of recurrence, about 21.5% at 2 years and 40.0–50.0% at 5 years after primary surgery (5). According to previous reports, the probability of recurrence would be greatly reduced for the patients receiving postoperative medication, which declined to 3–11% at 2 years and 6% at 5 years (6, 7). However, despite postoperative medication can effectively delay the recurrence, some patients still suffered recurrence more than 5 years after surgery. Unfortunately, few studies have analyzed the risk factors of endometrioma recurrence beyond 5 years after surgery. Simultaneously, there is no available prognostic model to predict the probability of postoperative recurrence of endometrioma more than 5 years follow-up after surgery. The most widely used American Society for Reproductive Medicine revised staging system (r-ASRM) staging has little effect on the prognosis of the disease. Therefore, it is necessary to establish a predicting model on endometriosis recurrence after surgery.

At present, nomograms have been widely developed in many kinds of diseases (8, 9). As a graphical representation of a mathematical model, nomogram consists clinical characteristics to predict a concerning event. By integrating various essential factors, a nomogram can provide evaluation of individual probabilities of certain disease, survival rates or recurrence after treatment of a benign or malignant disease (10). The representation of a nomogram model allows for outcome predictions more easily and quickly in clinical practice (11). Therefore, the nomogram has become an ideal tool for predicting the concerning clinical outcomes (9, 12–14).

Previous studies have shown that patients with endometrioma suffering from dysmenorrhea and/or adenomyosis were more likely to relapse, regardless of age at surgery, surgical strategies (cystectomy or unilateral ovarian excision), and weather combining postoperative medication (15). In this study, patients with unilateral or bilateral endometrioma who were younger than 45 years old at surgery, underwent cystectomy and were given postoperative medication (long-term or short-term) as well as completing long-term follow-up (≥5 years) were recruited to establish prognostic nomogram model. Based on the preoperative history and postoperative follow-up information, we attempt to establish a prognostic nomogram for endometrioma patients and determine whether the nomogram has the potential to predict the recurrence probability.

## Materials and Methods

### Patients' Inclusion

This study was conducted on a derivation cohort of patients with endometrioma who underwent laparoscopic cystectomy at Peking Union Medical College Hospital (PUMCH) from January 2009 to April 2013. The initial inclusion criterion included: age between 20–45 years old; the diagnosis of endometrioma was confirmed by pathologists; all patients were treated with postoperative medications such as gonadotropin-releasing hormone agonist (GnRH-a) injections for 3–6 cycles with or without a levonorgestrel-releasing intrauterine device (LNG-IUD), combined oral contraceptives (COCs) for at least 3 months, or LNG-IUD alone; ultrasonography was carried out to evaluate whether endometrioma recurrence at least 6 months after surgery; and the duration of postoperative follow-up was at least 5 years. The exclusion criteria included: age <20 or >45 years old; combined with ovarian malignant or borderline tumor; underwent uniliteral or bilateral oophorectomy or hysterectomy; and intraoperative conversion to laparotomy.

The last follow-up occurred in September 2018. This research was approved by the Ethics Committee of PUMCH, and written informed consent was obtained from all participants.

### Diagnosis, Treatment, Follow-Up, and Data Collection

The data for patients underwent cystectomy included preoperative, intraoperative, and postoperative follow-up information. Preoperative information of patients was collected as follows: age at surgery (divided into continuous five group), body mass index (BMI), gravity and parity, preoperative infertility, dysmenorrhea (Visual Analog Score, VAS, 0 = no dysmenorrhea, 1–4 = mild dysmenorrhea, 5–7 = moderate dysmenorrhea, 8–10 = severe dysmenorrhea, we combined the latter two groups into “moderate to severe dysmenorrhea”), dyspareunia, preoperative CA125 level, preoperative medication, medical history of endometrioma surgery. Intraoperative information, such as unilateral or bilateral cyst, the sum of both side cyst diameters (the size of the ovarian endometrioma was defined as the largest diameter of cysts, and the sum of left and right cyst was divided into ≤ 10 cm and >10 cm group), whether combined with uterine fibroids, adenomyosis, DE, as well as the r-ASRM stage. Postoperative follow-up information included postoperative therapy, pregnant condition, postoperative recurrence as well as the specific types of recurrence. Of note that, the different degrees of dysmenorrhea were determined based on previous related studies (16). In addition, we used imputation with mean, median or mode to replace missing data.

The patient was preliminarily diagnosed by medical history, gynecological examination, ultrasound examination and CA125 test before operation. The diagnosis was confirmed by surgery and histopathology. All surgeries were performed by one experienced laparoscopic surgeon team. In all cases, the aim of the surgical procedures was to perform lysis of adhesion, complete the opening of the pouch in the operation and remove all visible endometriotic lesions. Endometriosis was staged according to the classification of the r-ASRM. The presence, localization, and extent of typical powderburn and subtle lesions, adhesions, and deep infiltrating implants were recorded (15). After the endometriomas were removed, all remaining visible endometriotic lesions were excised or fulgurated (15). Anatomical restoration was then achieved. Specimens underwent detailed histopathological examination. In addition, for patients with uterine fibroids, surgeons performed myomectomy; for adenomyosis, biopsy or resection of the lesion was performed.

All patients received postoperative therapy. Short-term postoperative drug therapy refers to the use of drugs for <1 year, such as 3–6 cycles of GnRH-a treatment and short-term use of COCs ( ≤ 1year). Long-term drug therapy refers to the use of drugs for more than 1 year, such as the use of LNG-IUD alone or in combination with GnRH-a and long-term use of COCs (≥1 year). Pain recurrence refers to the recurrence of pain symptoms after at least 3 months of remission after surgery, having same or higher VAS score compared with preoperative level. Endometrioma recurrence was evaluated by transvaginal ultrasound, which defined as the presence of a persistent ovarian cyst with a thin wall (at least 2 cm in diameter), regular margins, a homogenous low echogenic fluid content with scattered internal echoes and did not resolve after several successive menstrual cycles (15, 17, 18).

### Statistical Analysis

The statistical analysis was performed using SPSS Version 22.0 (SPSS Inc., Chicago, IL, USA). Continuous variables were represented as mean ± standard (SD) or median with interquartile range (IQR), and categorical variables were showed as percentage. Quantitative variables were compared using *t*-tests or ANOVA, and qualitative were compared by Fisher's exact or chi-square tests. Univariate and multivariate analyses of Cox proportional hazards regression model were cooperated to determine recurrence related factors. During univariate Cox analysis, only those variables showing statistical significance (*P* < 0.1) or factors considered important in previous studies were included in a multivariate Cox proportional hazards regression model. According to the results of multivariate analysis, a nomogram was established by using R 4.0.0 software (Institute for Statistics and Mathematics, Vienna, Austria; http:// www.r-project.org/). The R library “rms” was used to build recurrence models. Discrimination refers to the predictive performance of the model. Predictive performance was assessed using the index of concordance (C-index), which resembles the area under the curve (AUC), but appears to be better suited for censored data (19). *C*-index will range from 0–1, and the closer it gets to 1, the greater predicting value of this Cox regression model (20, 21). As to model verification, it is better to use an external data set for model verification. If external data set is not available, bootstrap resampling methods based on internal data set and calibration curve will be recommended for validation (8, 10). The marginal estimate and the average prediction probabilities of the model were used to create a calibration curve. Calibration curve is to evaluate the consistency between the predicted probability and the observed probability. In a perfectly calibrated model, the prediction should fall on the diagonal 45° line of the calibration plot. To reduce the overfitting bias, the nomogram was subjected to 1,000 bootstrap resamples for internal validation in the training cohort (21). Furthermore, by “survival” and “survivalROC” libraries, the risk scores of patients based on developed model were calculated, and the AUC were calculated to access the predictive accuracy. In addition, the risk scores of patients based on developed model were calculated. Based on the risk scores, patients were divided into low-risk group and high-risk group. By using the dichotomized risk group as a factor, the survival curves were depicted by the Kaplan-Meier method and compared by log-rank test. A two tailed *P-*values <0.05 indicated statistical significance.

## Results

### Clinical and Laboratorial Characteristics of the Patients

A total of 323 patients with uniliteral or bilateral endometrioma who met the inclusion criteria were recruited in the derivation cohort. The clinical and laboratory characteristics of patients in the derivation cohorts are represented in Table 1.

**Table 1:** Demographic and clinical characteristics of patients with endometrioma.

**Demographic or characteristic**	**No. of patients**	**%**
Age (years), (M ± SD)	32.63 ± 0.29	
BMI (kg/m^2^), (M ± SD)	21.00 ± 0.14	
**Complaint**, ***n*** **(%)**		
Ultrasound-cyst	259	80.2
Dysmenorrhea	16	5.0
Dysmenorrhea-heavy	18	5.6
Infertility	12	3.7
Other symptoms	18	5.6
**Parity**, ***n*** **(%)**		
≥1	98	30.3
**Infertility**, ***n*** **(%)**		
Primary infertility	39	12.1
Second infertility	22	6.8
**Dysmenorrhea**, ***n*** **(%)**		
Mild (VAS 1–4)	44	13.6
Moderate-heavy (VAS 5–10)	208	64.4
Chronic pelvic pain, *n* (%)	54	16.7
**CA125 [*****n*** **(%)]**		
≤ 35 IU/ml	85	26.3
>35 IU/ml	238	73.7
Prior endometrioma surgery, *n* (%)	16	5
Preoperative GnRH-a treatment, *n* (%)	70	21.7
**Endometrial cyst localization**, ***n*** **(%)**		
Left ovary	94	29.1
Right ovary	80	24.8
Both side	149	46.1
**Sum of cyst diameters**, ***n*** **(%)**		
≤ 10 cm	244	75.7
>10 cm	79	24.5
Deep endometriosis, *n* (%)	177	54.8
Uterine fibroids, *n* (%)	57	17.6
Adenomyosis, *n* (%)	128	39.6
**r-ASRM stage**, ***n*** **(%)**		
I	1	0.3
II	8	2.5
III	102	31.6
IV	212	65.6
**Postoperative treatment**		
COCs, *n* (%)	12	3.7
COCs duration (months), median (IQR)	6 (4.5–12)	
LNG-IUD, *n* (%)	13	4.0
GnRH-a, *n* (%)	236	73.1
GnRH-a + LNG-IUD, *n* (%)	62	19.2
GnRH-a duration (months), median (IQR)	3 (3–3)	
LNG-IUD duration (months), median (IQR)	60 (45.75–60.0)	
**Postoperative pregnancy**, ***n*** **(%)**		
Natural pregnancy	104	32.2
Assisted reproductive technology	25	7.7
Natural abortion	3	0.9
Follow-up time (months), median (IQR)	84 (72–96)	
**Recurrence**, ***n*** **(%)**		
Ultrasound-Endometrioma	21	6.5
Endometriosis related pain	34	10.5
Both	10	3.1

*M ± SD, Mean and standard deviation; BMI, Body mass index; VAS, Visual Analog Scale; GnRH-a, Gonadotropin-releasing hormone agonist; r-ASRM, American Society for Reproductive Medicine revised staging system; IQR, Interquartile range; COCs, Combined oral contraceptives; LNG-IUD, Levonorgestrel-releasing intrauterine device*.

### The Recurrence of Endometrioma or Endometriosis-Related Pain

The median follow-up time was 84 (IQR, 72–96) months. At the end of follow-up, a total of 65 people (20.1%) relapsed, including 34 patients (10.5 %) with EM-related pain recurrence alone, 20 patients (6.5%) with endometrioma recurrence alone under ultrasound, and 10 patients (3.1%) with both recurrences.

### Independent Prognostic Factors in the Derivation Cohort

In the derivation cohort, Cox proportional hazard regression of univariate analysis and multivariate analysis were cooperated to screen potential risk factors. During the univariate Cox regression analysis, the following variables were selected for further multifactor regression analysis: (1) *P-*value <0.1, such as the sum of cyst diameters (*P* = 0.099), dysmenorrhea (*P* = 0.010), combination with uterine fibroids (*P* = 0.059), combination with adenomyosis (*P* = 0.035); (2) although the *P* >0.1, age (*P* = 0.168) was considered as important factors included for further multivariate analysis (divided into continuous five groups, 20–25, 26–30, 31–35, 36–40 and 41–45 years old). Finally, five variables were included into the multivariate Cox proportional hazard regression analysis: dysmenorrhea-degree, the sum of cyst diameters, combination with uterine fibroids, combination with AM, and age at surgery. Furthermore, multivariate analysis determined that the dysmenorrhea (dysmenorrhea-mild, HR = 5.329, 95%CI = 1.419–20.008, *P* = 0.013; dysmenorrhea-moderate to heavy, HR = 6.162, 95%CI = 1.904–19.946, *P* = 0.002), the sum of cyst diameters (HR = 2.067, 95%CI = 1.178–3.626, *P* = 0.011), presence of AM (HR = 1.799, 95%CI = 1.074–3.014, *P* = 0.026) and presence of uterine fibroids (HR = 1.780, CI = 1.012–3.128, *P* = 0.045) were independent risk factors for recurrence. The results of the multivariate analysis were listed in Table 2.

**Table 2:** Multivariate analysis of the derivation cohort.

**Variable**	**Recurrence**		
	***P***	**HR**	**95% CI**
Age	0.113	0.816	0.635–1.049
Dysmenorrhea-mild	0.013	5.329	1.419–20.008
Dysmenorrhea-moderate to heavy	0.002	6.162	1.9035–19.946
Sum of cyst diameters ≥10 cm	0.011	2.067	1.178–3.626
Uterine fibroids	0.045	1.780	1.012–3.128
Adenomyosis	0.025	1.799	1.074–3.015

### Prognostic Nomogram for Whole Recurrence

**Figure 1:**
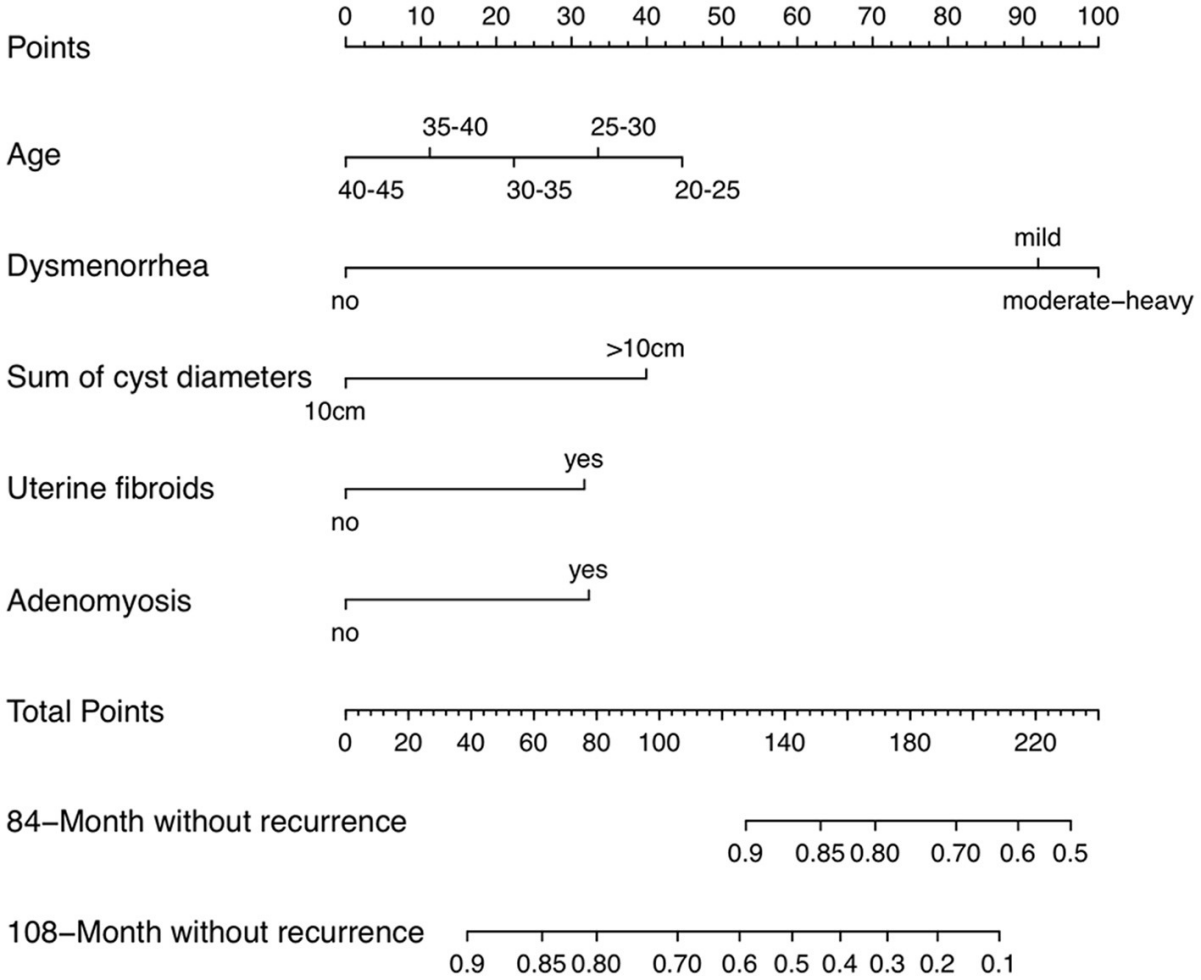
Endometrioma recurrence nomogram (To use a nomogram, you need to locate individual patient values on each variable axis, and then draw a line up to determine the number of points received for each variable value. The sum of these numbers is on the “total points” axis, and a line is drawn down on the survival axis to determine the probability of without recurrence of 84-month or 108-month).

The prognostic nomogram consisted of all the factors of the multivariate Cox regression model in the derivation cohort. It established scoring criteria in term of the hazard ratio (HR) values of all prognostic factors. Every prognostic factor had a corresponding score. Then according to a certain proportion, the line segments with scale were drawn on the same plane and displayed in a graphical way. The prognostic nomogram for 84- and 108-month without recurrence was shown in Figure 1. By adding the scores corresponding to each variable and projecting the total score to the bottom scale, the probabilities of without recurrence for 84- months and 108- months could be estimated. With the help of nomogram, the prognosis could be effectively predicted based on the patient's personal characteristics.

### Validation of the Nomogram

**Figure 2:**
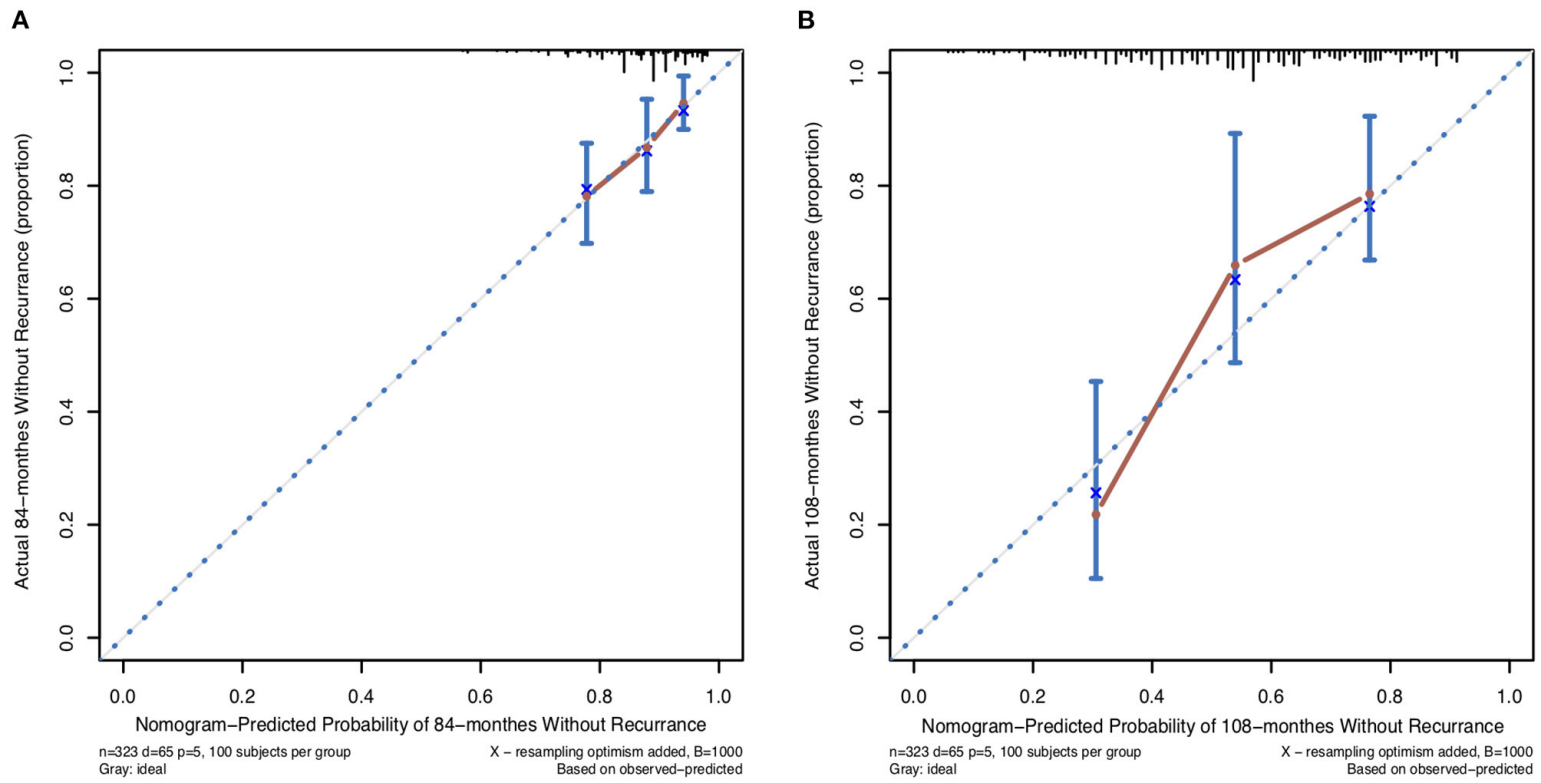
The calibration curve for predicting patients without recurrence at **(A)** 84-month and **(B)** 108-month in the derivation cohort. Nomogram-predicted probability of overall without recurrence is plotted on the x-axis, actual overall without recurrence is plotted on the y-axis.

**Figure 3:**
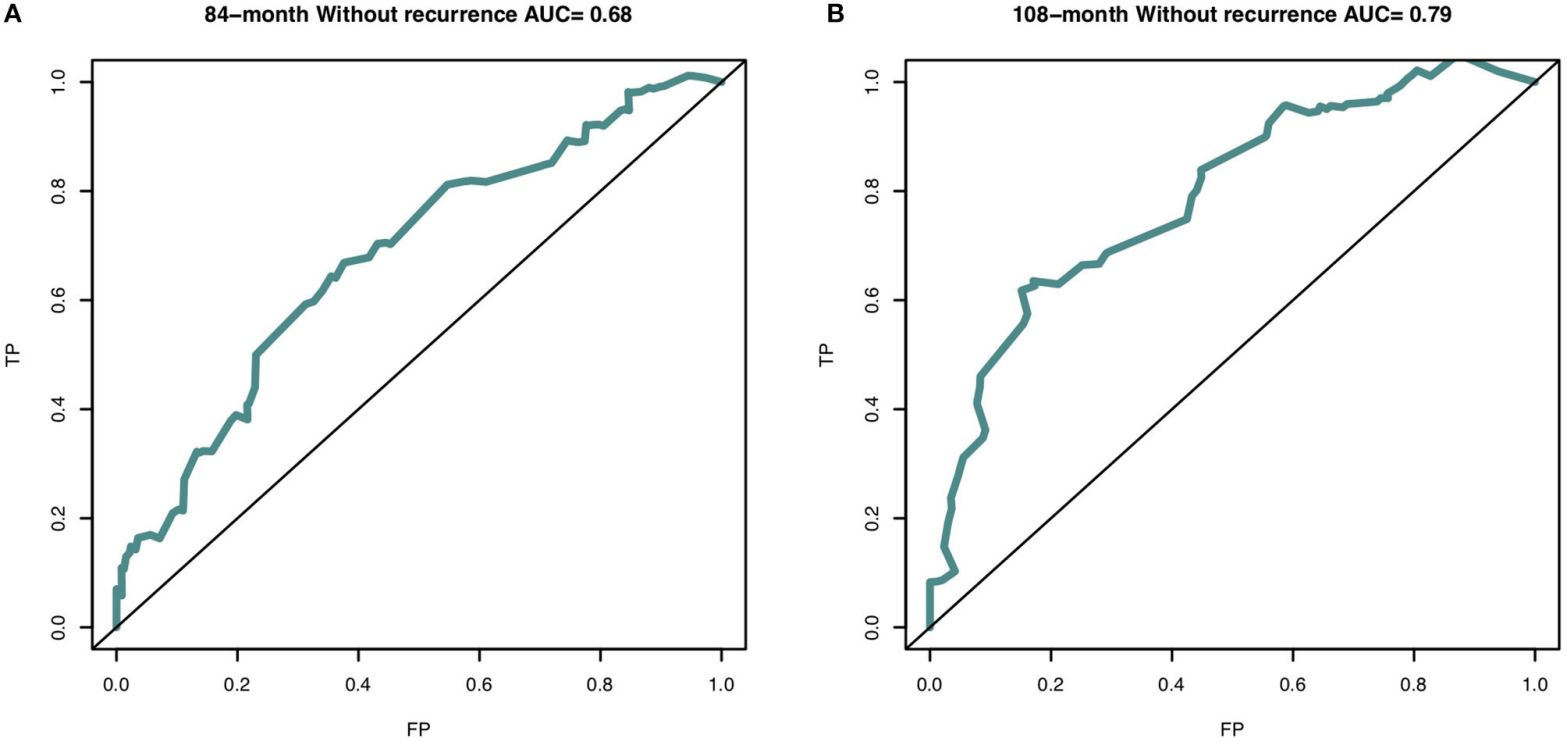
The area under curve (AUC) of nomogram predictive model at **(A)** 84-month (AUC = 0.716) and **(B)** 108-month (AUC = 0.796) in the derivation cohort. The receiver operating characteristic curve (ROC) is made via R package “survivalROC”. TP, true positive-rate; FP, false positive-rate.

**Figure 4:**
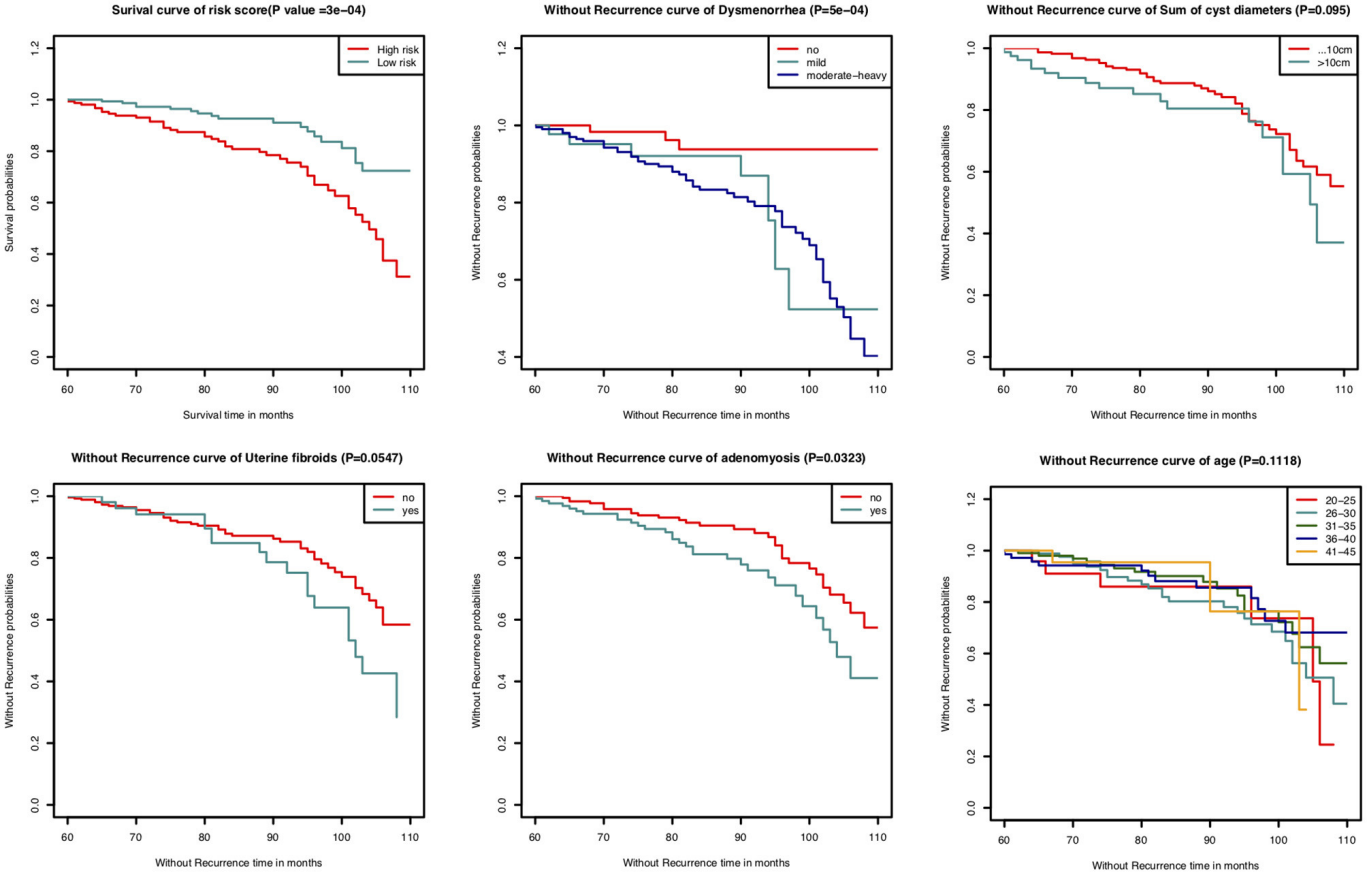
Kaplan-Meier survival curves of derivation cohort.

In this study, the C-index for without recurrence prediction was 0.683 (95% CI, 0.610–0.755). The calibration curve for the probability of without recurrence after surgery showed an optimal agreement between the predicted and observed values for 84- and 108-month recurrence in the derivation cohorts (Figures 2A,B). In addition, according to the Cox proportional hazard regression model, the risk scores of all patients were calculated. And according to the risk scores and the observed recurrence results, the AUC for 84-month without recurrence and 108-month without recurrence were 0.680 and 0.790, respectively (Figures 3A,B). The Kaplan-Meier method showed that there was a statistically significant difference in recurrence probability between the low-risk group and the high-risk group (*P* < 0.05) (Figure 4). The independent factors in the model, such as dysmenorrhea degree and presence of AM were significantly different in the Kaplan-Meier single factor survival analysis (Figure 4).

## Discussion

The nomogram is a graphical representation of a mathematical model that combines biological and clinical variable to determine the probabilities of clinical events. With the feature of convenience, simplicity and practice, nomogram is widely used for predicting the prognoses in patients with benign or malignant disease. In our study, by analyzing the preoperative, intraoperative, and postoperative follow-up information, we can predict the recurrence probability of EM and/or EM-related pain for endometrioma patients over long-term follow-up after surgery, in order to screen high-risk patients for additional intervention and treatment.

EM is an easy recurrent disease after surgery. Postoperative therapy can significantly reduce the probability of recurrence. Common medication treatments included COCs, GnRH-a, LNG-IUD, and dienogest. These drugs can be used alone or in combination. However, Jee et al. has an idea that the drug treatment did not prevent the EM from recurrence, but only prolonged the interval after surgery (18). In addition, different drugs have different duration of use. GnRH-a is usually used for a short period of 3 to 6 months, while LNG-IUD mostly takes effect for up to 5 years. The use time of COCs is relatively flexible, shorter than or more than 1 year. In our study, 298 (283/323, 92.3%) patients received GnRH-a treatment after surgery and the median duration was 3 (IQR, 3-3) months. 75 patients underwent LNG-IUD treatment, of which 62 patients were pretreated with GnRH-a firstly. The median medical duration of patients who received COCs was 6 (IQR, 4.5–12) months (Table 1). Therefore, although all patients have accepted drug treatment after surgery, most of the treatment was short-term, which was partly attributed to pregnancy desire. 75 patients received LNG-IUD treatment, of which 72 patients who recorded the medication time had a median time of 60 (IQR, 45.75–60.0) months. There were 51 patients who had been treated with LNG-IUD for 5 years. The main reasons for interrupting LNG-IUD treatment included poor effect or ring shedding (5 patients), side effects (5 patients), perimenopause or menopause (5 patients), pregnant desire (4 patients) as well as other unknown reasons. In our follow-up data, most patients chose to use GnRH-a alone for 3 cycles after operation. Only patients using LNG-IUD and a few patients taking COCs received medication for more than 1 year. In patients who have been using LNG-IUD for up to 5 years, we also did not observe significant protective effect in the Cox regression analysis. There was selection bias in patients, because we found that patients who use LNG-IUD have a higher probability of concurrent adenomyosis (*P* < 0.001), of which patients tended to have more severe symptoms. According to the nomogram, specific management strategies can be established for patients with high-risk recurrence. A systematic review concluded that long-term postoperative hormonal treatment had a protective effect for prevention of endometrioma recurrence after ovarian cystectomy in cohort evidence (22). Even if postoperative medication does not reduce but merely delay the recurrence of the disease, it may be an option for patients at high risk of recurrence to extend the duration of drug therapy to delay the recurrence of the disease until the perimenopause.

Nowadays, EM has been regarded as a chronic disease (23). In addition to emphasizing the therapeutic effect of surgery, preventing disease progression and postoperative recurrence, we also emphasize the need for long-term drug management of EM (24). Combined with the nomogram model developed in this study, for patients who still have a high risk of recurrence even received postoperative medication, the duration of medication should be extended. During long-term follow-up, the treatment protocol should be adjusted in time according to the condition. Although the patients in this study were treated with drugs after surgery, most patients received short-term postoperative therapy. In this case, we were concerned about the cumulative recurrence risk of patients at 84 and 108 months after surgery. Even after the operation, patients were treated with LNG-IUD, and the efficacy would gradually disappear without replacement in time. Therefore, this predictive nomogram can further emphasize the importance of long-term management of postoperative therapy, especially for patients with high risk of recurrence.

In the present study, the nomogram was established based on 323 endometrioma patients in China and used to predict the 84 and 108 months of recurrence probability. According to previous studies, age at surgery has been demonstrated to be associated with postoperative recurrence of endometriosis, and particularly, 35 years old is considered as a cut-off point. Patients with endometrioma who younger than 35 years old are more likely suffering from recurrence after surgery (25). In our nomogram, age at surgery was also included in the model to predict the probability of postoperative recurrence.

This study had some limitations. Firstly, the prediction ability of the nomogram is relatively accurate, but the probability of the terminal event is low. One of the most important reason is that we only enrolled 323 patients and all of them received either short-term or long-term medical treatment after surgery. Therefore, after 60–120 months of follow-up, only 65 patients suffered recurrence, which might be figured out by expanding the sample size or extend the follow-up time. Secondly, the nomogram was developed based on data of a single institution in China and no available ideal external data set for verification. Therefore, it is necessary to expand the sample size, and larger cohorts across multiple centers are needed to further evaluate the clinical utility of our nomogram in the future. Thirdly, although the C-index of the nomogram was good, it was not excellent. Many other factors may influence the prognosis. In addition, the recurrence outcomes should be analyzed separately from endometrioma recurrence and EM-related pain recurrence, because the recurrence of EM-related pain cannot exclude the recurrence of adenomyosis, especially for patients with a history of adenomyosis. In this study, due to the limitation of the sample size, EM-related pain did not rule out the combination with adenomyosis, because many studies regarded adenomyosis as a special type of EM or thought them enjoyed the same etiological factors, and these two diseases often occur together (23). The pathogenesis and drug treatments of them are very similar. The premise of solving this problem is to solve the first two limitations.

## Conclusion

We developed a predictive nomogram to predict the probability of no recurrence within 84 and 108 months in patients with endometrioma who received surgery and postoperative drug treatment under the age of 45. The proposed nomogram provided statistically significantly better discrimination and offered a useful tool for prognosis. In addition, to further polishing the nomogram model, expanded sample size, extended follow-up duration, and additional validation with external data are required.

## Data Availability Statement

The datasets used and/or analyzed during the current study are available from the corresponding author on reasonable request.

## Ethics Statement

Peking Union Medical College Hospital Ethics Committee approved this study (Ethic trial No. JS-1532) and all patients were informed of the study and provided consent. Written informed consent was obtained from the individual(s) for the publication of any potentially identifiable images or data included in this article.

## Author Contributions

XL and JL developed the idea for the project and critical discussion. XL and ZG designed the study. ZG performed the data analysis and takes full responsibility for the integrity of the data. JZ, YW, CZ, HY, and ZG collected the medical data and drafted the manuscript inputs. All authors contributed to the article and approved the submitted version.

## Funding

This work was supported by the National Key R&D Program of China (No. 2017YFC1001200) and the National Natural Science Foundation of China (No. 82071628).

## Conflict of Interest

The authors declare that the research was conducted in the absence of any commercial or financial relationships that could be construed as a potential conflict of interest.

## Publisher's Note

All claims expressed in this article are solely those of the authors and do not necessarily represent those of their affiliated organizations, or those of the publisher, the editors and the reviewers. Any product that may be evaluated in this article, or claim that may be made by its manufacturer, is not guaranteed or endorsed by the publisher.

## Abbreviations

EM: endometriosis
PEM: peritoneal endometriosis
DIE: deep infiltrating endometriosis
r-ASRM: American Society for Reproductive Medicine revised staging system
GnRH-a: gonadotropin-releasing hormone agonist
LNG-IUD: levonorgestrel-releasing intrauterine device
COCs: combined oral contraceptives
BMI: body mass index
VAS: Visual Analog Score
AUC: area under the curve.
